# Comprehensive review on the potential of ultrasound for blue food protein extraction, modification and impact on bioactive properties

**DOI:** 10.1016/j.ultsonch.2024.107087

**Published:** 2024-09-27

**Authors:** Ghumika Pandita, Savvy Sharma, Irin Elsa Oommen, Nishchhal Madaan, Yuvraj Bhosale, Vivien Nagy, Ayaz Mukarram Shaikh, Béla Kovács

**Affiliations:** a Department of Food Technology and Nutrition, School of Agriculture, Lovely Professional University, Phagwara, Punjab, India; b Punjab Agricultural University, Ludhiana, India; c Research Engineer, Indian Institute of Technology, Kharagpur, India; d Faculty of Agriculture, Food Science, and Environmental Management, Institute of Food Technology, University of Debrecen, Böszörményi út 138, 4032 Debrecen, Hungary; e Faculty of Agriculture, Food Science, and Environmental Management, Institute of Food Science, University of Debrecen, Böszörményi út 138, 4032 Debrecen, Hungary; f Doctoral School of Nutrition and Food Sciences, University of Debrecen, Böszörményi út 138, 4032 Debrecen, Hungary

**Keywords:** Blue protein, EAAs (Essential Amino Acids), Power ultrasound, Bioactive peptides, Anti-hypertensive activity

## Abstract

Food security for the increasing global population is a significant challenge of the current times particularly highlighting the protein deficiencies. Plant-based proteins could be considered as alternate source of the protein. The digestibility and PDCASS value of these proteins are still a concern. Blue proteins, the new approach of utilizing the proteins from aquatic sources could be a possible solution as it contains all the essential amino acids. However, the conjugation of these proteins with fats and glycogen interferes with their techno-functional properties and consequently their applicability. The application of power ultrasound for extraction and modification of these proteins from aquatic sources to break open the cellular structure, increase extractability, alter the protein structure and consequently provide proteins with higher bioavailability and bioactive properties could be a potential approach for their effective utilization into food systems. The current review focuses on the application of power ultrasound when applied as extraction treatment, alters the sulphite and peptide bond and modifies protein to elevated digestibility. The degree of alteration is influenced by intensity, frequency, and exposure time. The extracted proteins will serve as a source of essential amino acids. Furthermore, modification will lead to the development of bioactive peptides with different functional applications. Numerous studies reveal that blue proteins have beneficial impacts on amino acid availability, and subsequently food security with higher PDCAAS values. In many cases, converted peptides give anti-hypertensive, anti-diabetic, and anti-oxidant activity. Therefore, researchers are concentrating on ultrasound-based extraction, modification, and application in food and pharmaceutical systems.

## 1 Introduction

Globally, an increasing need for high-quality protein has become evident. The conventional sources of the proteins take many resources for their production making it difficult to cope with the global population increase and protein demand. The utilization of unconventional sources has the limitations such as complex protein structure, conjugation with other macro and micronutrients, variable functionalities and lowed bioavailability hampering their utilization. Aquaculture, the practice of raising, breeding, and harvesting aquatic organisms, is currently the fastest-growing food production sector globally and a sustainable option for attaining food security. Owing to its unique role, it is expected that the demand for aquatic products (especially seafood) will continue to increase due to geometric population growth [1]. Blue foods, a recently popularized nonconventional food, commonly referred to as aquatic foods either from plant source or animal source, assume a pivotal role in ensuring food and nutrition security [2]. Invertebrates, algae, and aquatic plants which are harvested or cultivated in freshwater and marine environments, can be categorized as blue foods that offer a viable way towards meeting this demand. Frequently disregarded in nutritional discussions, blue foods also comprise a wide range of lesser-known consumable sources, including unusual botanicals like purple potatoes and blueberries [3].

The protein derived from blue foods can serves as a vital and sustainable resource, playing an irreplaceable role in meeting the ever-growing global demand for food with increased population. Blue foods offer a promising alternative as a source of protein compared to terrestrial livestock due to their rich content of healthy fats, including Omega-3 fatty acids, which are known to mitigate obesity and non-communicable diseases [4]. The inclusion of Omega-3 fatty acids further enhances the nutritional value of protein derived from various fish sources [5].

Despite having a higher moisture content, blue foods exhibit a higher protein content on an edible fresh weight basis compared to most terrestrial protein sources [5]. Additionally, the superior digestibility and high biological value of proteins derived from blue foods renders it a valuable source in comparison to animal-derived protein sources [4]. Integrating blue foods into diets can effectively address protein deficiencies, particularly in regions with limited access to conventional protein sources. Moreover, the environmentally sustainable and nutrient-rich production methods associated with blue foods, such as spirulina, present an opportunity to tackle challenges related to global food security [6]. Therefore, it is imperative for aquatic food systems to uphold biodiversity and ecological services while ensuring the abundant production of nutritious and secure food crops [7]. For instance, aquatic plants, notably seaweeds prevalent in the diets of the Asia-Pacific region, serve as an exemplary source of both nutrition and a low-carbon food option [8].

Food security determines one's access to foods required to meet nutritional requirements and hence, directly influences the expected quality of life [9]. Multiple forms of malnutrition are still prevalent and universal today at a global scale. Among children under the age of five, 149 million (22 %) are affected by stunting and 45 million by wasting [5]. Blue foods offer a promising solution to address these alarming concerns. However, shortfalls present in the current food processing techniques of blue foods, hinders this opportunity. There are several setbacks in the processing of aquatic foods which include losses due to mishandling, disease and quality loss. Short supply of labour coupled with a high turnover is a contributing factor [10]. Majority of the FLW (Food Loss and Waste) occur at the household level due to shorter shelf lives of aquatic foods. Slimy appearance and odours account for over 6 % of wasted aquatic food [11].

To utilize these nonconventional sources, the protein can be extracted and modified for desirable functionalities and the aforementioned drawbacks can be overcome by employing ultrasound processing on aquatic foods, which specifically plays a crucial rule in protein extraction. This highlights the necessity for sustainable and effective extraction and processing techniques. High-frequency ultrasound waves can inhibit microbial growth, delaying spoilage, and reducing the need for chemical preservatives [12]. Furthermore, blue foods like blue-green algae can yield maximum amounts of minerals and bioactive substances when extraction is done using ultrasound-assisted extraction techniques. When applied with high power ultrasound, the waves cause bubble formation that undergoes cyclic compression and rarefaction in further treatment. Due to progression in the treatment, the cyclic process results into the bursting of the bubble creating a very high localised temperature and pressure. This pressure–temperature combination causes disruption of cell structures, in-turn releasing proteins, vitamins, and antioxidants [13]. Recognizing the fact that food security is not constant and that the stability of the food supply by utilizing such techniques is also an important dimension of food security is essential [9]. Harvesting aquatic foods and extracting their proteins using ultrasound techniques can hence, address these challenges and provide both nutrition and economic stability.

Protein extraction can be explained as bringing the protein into solution by breaking the tissue or cells or extracting them from various sources. Extraction of proteins such as bioactive peptides from microalgae, are recovered through conventional alkaline extraction [14]. Additionally, sequential extractions using multiple technological approaches have been successfully used to improve the extraction of high-value compounds from aquatic sources [15]. However, these conventional techniques are time consuming, produces lower yield and generate higher waste content [16]. Hence, we can use ultrasound processing as a novel technique for protein extraction. [16] observed that microalga *Chlorella vulgaris* exhibited high protein recovery when US-assisted extraction was done. This was enabled by utilizing alternating compression and rarefaction waves. An almost complete protein recovery of mackerel fish was found possible using the same method [1]. As understood from the given examples, US technology proves to be a legitimate option for protein extraction from blue foods with an undeniable role in assuring food security [2].

The application of ultrasound technology is well underlined for the extraction of proteins along with their modifications. However, the literature stating its utilization in the blue foods and protein extraction and modification is limited. The current review is aimed to aggregate the information to explore blue foods as an alternative protein source, examining their types as well as quality of blue foods known to us. Further, the aim is to focus on the comparative advantages of utilizing US techniques to facilitate protein extraction and establish the relationship between US and modification in protein structure is studied and explored the development of bioactive peptides. The significance of utilizing bioactive peptides derived from blue foods is exemplified as well. Ultimately, disseminate the we expound the impact of such advancements on various sectors and thereby, aim to understand the future prospects of utilizing blue foods as a sustainable source of protein.

## 2 Blue foods as source of protein

Proteins are essential macronutrients which we require for sustained human health. We achieve our required intake from a variety of sources, mainly animal based foods. “Blue foods” such as fish and crustacean animals are exceptional sources of protein. Their nutritional value can be contributed to the presence of omega-3 and fatty acids, aside from the protein content available [17].

### 2.1 Types of blue foods

Food proteins are highly important in providing essential amino acids required for sustenance of the human body. As mentioned, fish and crustaceans are referred to as “blue foods” due to their origin from oceans along with their high nutritional value. For instance, fish is a substantial source of nutrition in Southeast Asia and Africa, especially in the preserved forms such as dried, salted, fermented and smoked [18]. Marine fishes such as *Stolephoruswaitei, Stolephoruscommersonii* and *Rastrelligerkanagurta* are rich in glycine and glutamic acid besides leucine. The cold water species such as salmon, tuna and mackerel, are rich in lysine and aspartic acid, marine fishes in leucine, small indigenous fishes in histidine, and the carps and catfishes in glutamic acid and glycine [19].

Similarly, seaweeds and microalgae are considered a viable source of protein. The essential amino acid composition meets FAO (Food and Agriculture Organization) requirements and are often in par with other protein sources such as soybean and egg. The distinctive ‘umami’ taste in seaweed is associated with presence of aspartic and glutamic acid. Spirulina species of microalgae contains high protein content of 63 %. It is the most highly consumed microalgae. Seaweeds may be traditionally consumed as a sushi wrap and incorporated into noodles, breads, biscuits, sweets and beer [20].

Shellfish is also an excellent blue food protein source. The development of shellfish aquaculture has an important role in sustainable food supply and food security [1]. Shellfishes are essentially the aquatic invertebrates possessing a shell or exoskeleton [21]. They consist of molluscs and/or crustaceans, such as mussel, clam, crab, lobster, shrimp or prawn, etc. shellfish is a low-fat, low-saturated-fat, high-protein food that contains all the dietary-EAAs for the human body. They contain appreciable quantities of digestible proteins, EAAs, bioactive peptides, long-chain polyunsaturated fatty acids, astaxanthin and other carotenoids, vitamin B_12_ and other vitamins. They are rich in important minerals, including copper, zinc, inorganic phosphate, sodium, potassium, selenium and iodine [22].

### 2.2 Quality of blue food proteins

Proteins are vital macronutrients comprising EAAs, which play a crucial role in maintaining human health. Examining the EAAs composition, digestibility, and PDCAAS (Protein Digestibility Corrected Amino Acid Score) value of proteins obtained from blue foods such as fish, blueberries, spirulina, and blue corn is crucial to determining the quality of these proteins [23]. Aquatic animal proteins are rich dietary sources of methionine and lysine [19]. The extent of protein digestibility determines the extent of adequate utilization of amino acids for supporting health and immune system. In this context, amino acid bioavailability can be defined as the proportion of amino acids reaching systemic circulation and that can be incorporated into body protein synthesis [24]. Digestibility of protein by enzymes depends on several factors but mainly depends upon the type of raw material. Further, it depends upon the form of protein, amino acid chain and branching with other compounds like carbohydrates [25].

Considering tilapia fish as a protein source, the protein content in tilapia is 18.7 % [26]. In a study by Susanti et al., 2021, the ratio of EAAs to lysine (=100 %) was as follows: arginine (80.73); phenylalanine (69.73); histidine (34.15); isoleucine (51.24); leucine (66.01); methionine (41.49); threonine (88.96); tryptophan (23.07) and valine (72.63), suggesting rich sources of protein crucial for human health. Protein digestibility of tilapia is generally less, but varies depending on heat treatments applied. Heating causes protein denaturation which increases protein digestibility [26]. Furthermore, digestibility decreases when Maillard reaction occurs during heating and with application of higher heat temperatures between 180 to 300 °C, with an average value of protein digestibility (%) of 88 %. The PDCAAS value of tilapia is given in Table 1 [27].

**Table 1:** The source of protein in blue food.

Source of Protein	Type of Protein	Essential amino acid	Protein digestibility	PDCAAS value	Reference
Mollusc	Collagen, Shell matrix protein	Aspartic acid, Glutamic acid, Glycine, Alanine and Leucine	14.37 ± 1.03	0.69–0.85	[33]
Tilapia	Lean protein	Lysine, Methionine, Arginine, Phenylalanine	18.7 %	1	[34]
Cod	Lean protein	Methionine, Valine, Histidine, Lysine, Tryptophan	81.40 % to 82.19 %	0.96	[35], [36]
Tuna (Pacific Bluefin tuna)	Myofibrillar proteins, Sarcoplasmicproteins	Histidine, Isoleucine, Leucine, Lysine, Methionine, Phenylalanine, Threonine, Tryptophan.	68.4 %	0.97	[37]
Salmon	Complete protein	Methionine, Threonine, Valine	85 %	1	[38]
Herring	Lean protein	Lysine, Leucine, Threonine	80 %	1	[39]
Mackerel	Complete protein	Glutamic acid, Cysteine, Glycine, Aspartic acid, Alanine, Proline, Arginine	96.99 %	1	[40], [41]
Trout (Rainbow trout)	Lean protein	Glutamic acid, Aspartic acid, Arginine, Leucine, Lysine	51.5 % to 66.7 %	0.998	[42], [43]
Cuttlefish (mollusc)	Myosin	Histidine Isoleucine Leucine Lysine Methionine Phenylalanine Threonine TryptophanValine	85.4 %	70.9	[44]
Octopus	Lean protein	Arginine, Leucine, Lysine, Phenylalanine, Threonine, Isoleucine, Methionine	86.2 %	77.6	[44]
Red Seaweed (*Palmaria palmate*)	Phenolic compounds, Phycobiliproteins, Lectins	Leucine, Valine, Threonine, Phenylalanine	0.78 ± 0.002	0.69 ± 0.014	[45]
Water Spinach	Crude protein	Leucine, Tyrosine, Lysine, Threonine	73.8–77.9 %	0.93	[46]
Squid	Myofibrillar protein	Threonine, Arginine, Methionine, Valine, Phenylalanine, Leucine, Isoleucine, Lysine, Histidine.	93.2 %- 97.68 %	1	[47]
Mussel	Polyphenolic proteins	Valine, Leucine, Histidine, Methionine, Isoleucine, Lysine, Tyrosine, Tryptophan, Threonine, Arginine, Phenylalanine.	>90 %	0.93	[48]
Crab	Lean protein	Leucine, Lysine, Isoleucine, Phenylalanine, Tryptophan, Valine, Methionine, Arginine, Histidine, Threonine.	66 %	1	[49]
Green seaweed (Ulva species)	Phycobiliproteins, peptides	Leucine, Valine, Threonine, Phenylalanine, Lysine, Methionine	68.13 % Or 0.79 ± 0.003	0.15 ± 0.014	[45], [50]
Brown seaweed (Fucusserratus)	Phycobiliproteins, amino acids, lectins	Leucine, Valine, Threonine, Phenylalanine	0.77 ± 0.001	0.63	[50]
Shrimp	α-amino acids	Arginine, Methionine, Valine, Threonine, Isoleucine, Leucine, Lysine, Histidine, Phenylalanine, Tryptophan.	>90 %	1.0	[21]
Lobster	Glycine, Glutamic Acid, Proline	Valine, Leucine, Isoleucine, Methionine, Histidine, Threonine, Lysine, Arginine.	65–86 %	0.95	[51]
Sardines	Complete protein	Histidine, Leucine, Lysine, Valine, Methionine, Tryptophan	93–95 %	0.95	[52], [53]
Anchovies	Leucine, Glutamic acid, aspartic acid	Valine, Leucine, Threonine.	85 %	1	[54], [55]
Scallop	Taurine, Glycine, Actin, Promyosin	Cystine, Tryptophan, Isoleucine	80.2 %	0.67	[56]
Cockles	Lysine, Arginine	Taurine, Glutamic acid, Lysine, Arginine	80 %	97 %	[57]
Brown Seaweed (*Alariaesculenta*)	Phenolic compounds, Phycobiliproteins, Lectins	Leucine, Valine, Threonine, Phenylalanine	0.78 ± 0.002	0.59 ± 0.021	[45]
Red Seaweed (*Asparagopsistaxiformis*)	Glutamic acid, Proline, Glycine	Leucine, Valine, Threonine, Phenylalanine	0.79 ± 0.001	0.393 ± 0.01	[45]

Wong & Cheung, (2001) conducted a comprehensive nutritional evaluation of seaweeds, shedding light on their potential as a valuable food source. One notable finding was the relationship between in vitro protein digestibility and the total phenolic content of seaweeds. As reported, an increase in the total phenolic content led to a decrease in in vitro protein digestibility. This suggests that the presence of phenolic compounds in seaweeds can influence the efficiency of protein digestion [28]. The digestion of seaweed proteins involves the action of various proteolytic enzymes such as pepsin, pancreatin, pronase, trypsin, and chymotrypsin [29], [30]. (Fleurence, 1999; Fujiwara-Arasaki et al., 1984) Notably, the in vitro protein digestibility varied among different seaweed species, with the Hypnea and Ulva PCs (Protein Concentrates) displaying ranges between 85.7 % and 88.9 %. Interestingly, red seaweed exhibited higher in vitro protein digestibility compared to green seaweed [28].

Seaweed is rich in leucine, valine and threonine. The amount of EAAs of the seaweed PCs accounted for 36.2 ± 40.2 % of total amino acid content. Except for the sulphur containing amino acids (methionine and cysteine) and lysine, the levels of all the EAAs are higher than those of the FAO/WHO requirement pattern [31]. Furthermore, no cysteine is present in any of the seaweed PCs. The total amino acid content (ranged from 73.9 to 78.7 g/100 g PCs) of each seaweed PCs is comparable to their corresponding % protein (76.3 ± 85.0 %). This indicates that there are no non-protein nitrogenous materials in seaweed PCs. The chemical score (CS) is 57 %. Seaweed has a PDCAAS value of 0.43 [32]. However, numerous protein sources along with their quality are stated in Table 1.

## 3 Protein extraction using ultrasound

Proteins are essential bio-molecules that are present in a wide variety of aquatic creatures and have a wide diversity of shapes and compositions. Understanding these proteins' distinct structures, their interactions with other bio-molecules like lipids and glycogen, and the application of effective extraction techniques are necessary for removing these proteins from various sources. With benefits like shorter extraction times, higher yields, and less disruption to protein structures, ultrasound-assisted protein extraction has become a viable method. The protein structural components of various aquatic species, including fish, shellfish, and marine algae, possess complex arrangements that interact with other molecules within the protein matrix [58]. This review delves into the impact of ultrasound-assisted extraction (UAE) methods and exploration of such complex structures. These intricate proteins are essential for the physiological processes of these organisms including their survival, showcasing a diverse array of cellular actions and their potential practical activities. The texture and nutrient composition of flesh are greatly influenced by the variety of proteins present in the fish. Myofibrillar, connective tissue and sarcoplasmic proteins plays the crucial role in enhancing nutritional composition to the fish’s flesh. Similarly, marine algae rely on proteins for numerous functions such as formational stability with defensive mechanism. Furthermore, shellfish depend on proteins for potential growth of shells and for the muscular contractions [59].

The crucial components of myosin and actin in relation to the activity and structural composition of muscles are predominantly situated within the protein matrix of aquatic organisms. The organized arrangement of these proteins enables intricate filament formation, notably within their musculature and serves as a structural base for locomotion). This is evident to the rapid propulsion abilities of fish as a result in the prevalence of myofibrillar proteins in their skeletal muscles. On the other hand, octopuses and squid, revered for their soft-bodied nature as molluscs, possess a unique protein arrangement composed of collagen-like proteins [60]. Meanwhile, fish − an abundant source of aquatic proteins, consist of numerous muscular amino acids. These proteins include myoglobin and metabolic enzymes that assist in sustaining cellular functions and preserving the pigment, along with actin and myosin activity which builds up the contractile foundation known as myofibrillar proteins. The two imperative proteins such as collagen and elastin present in the connective tissue enhance the fish structural stability. The protein found in shellfish, consists of potential structure stability along with array of enzymes that boosts up the muscle contraction. The protein act as defensive mechanism, vital for the construction of shells and develop an efficient immune system. Exemplifying this are the algal lectins and phycobilisomes, which serve as prime examples of structural constituents found in marine algae along with other photosynthesis-involved proteins like phycobiliproteins [61].

Aquatic creature's protein structure is spread out across a complicated matrix involving other bio-molecules. In fish, for instance, fats and proteins exist together which enhances the taste and boost its nutritional aspects. Mussels have glycogen mixed with proteins to expand energy and the vitality for maintaining its muscle functions. In the cell wall, polysaccharide and lipids along marine algae pigments fuses together in the presence of proteins and create a fused −complex matrix. The actions among this matrix's elements impact how steady and useful proteins are with this interactivity. With the presence of fat and glycogen, the protein matrix works as a place for storing energy. Fat, also known as lipids, can be found in varying quantities across various aquatic species [62]. The hepatopancreas also known as digestive gland of crustaceans and molluscs may contain lipids. These are mainly for the metabolic needs and reproduction support. Another element that makes up the protein matrix of aquatic species is a complex carbohydrate termed as glycogen. During the condition of energy bursting, the requirement of fast energy supply is worked up by glycogen. Glycogen acts as an immediate energy reserve in muscles which is mainly crucial for those fish-types that quickly swims [63].

Organisms living in water can create and absorbs glycogen within special cell units. During the period of migration or during fleeing from the predators, maintaining high energy needs is crucial. Also, the framework of protein found in animals that reside in water usually contains essence of vitamins, minerals and enzymes. Some aquatic animals are mineralized with elements such as calcium and phosphorus. These macro-minerals assist to form bone and skeletal formation which helps the aquatic mammals in movements such as swimming or diving into depths. Vitamins, found in aquatic species are required for many metabolic operations such as to develop, reproduce and maintain good health. Biological catalysts such as enzyme, helps in the physiological functions through the biochemical reactions which help in digestion process as well as other metabolism [64]. In aquatic creatures, enzymes for digestion assist in the splitting of complex to simpler food parts for absorption and utilization. The arrangement of these protein components might differ based on aquatic species, respective habitat along with ecological place where the water body was residing. The unique protein compositions of numerous creatures living in water are affected by different factors such as diet, environment around them and changes due to evolution [65].

Protein extraction aided by ultrasound has been noticed for its ability to break the complicated protein matrix effectively. The major advantage of this process is to obtain high yield of extract within a shorter time and has lesser harmful effects on the structure of protein. In the process of extracting proteins with ultrasound, the structural cells are disrupted of the proteins in order get released out. The creation and breaking of microscopically small bubbles in tissue, known as cavitations, is made possible by ultrasound waves. This kind of disturbance assists in the disintegration of cellular membranes which leads to the freeing up of proteins, lipids and other elements inside a cell [66]. The use of sonication through ultrasound has been proven to be an effective method for extracting proteins. Sonication using ultrasound method leads to break down cellular structures precisely with high efficiency without causing fatal effects and harm on the extracted protein materials [66]. Ultimately, the protein build-up of aquatic animals is varied and intricate, serving specific activities and physiological functions. Conceptualizing the proteins distribution, interactions with lipids, enzymes, glycogen and macro-minerals results out about the aquatic organism’s biochemistry. Methods to extract proteins using ultrasound are helpful in studying proteins and utilizing them for many scientific as well as industrial purposes [67].

Ultrasound-assisted protein extraction and separation employ sound waves of high frequency to dismantle cellular structures. This aids in the liberation and individualization of proteins from organic matter. When ultrasound waves move through a liquid medium, they create cycles of high pressure and low pressure. These cycles generate cavitations which form small bubbles that grow rapidly and then explode out. In the solution, production of micro streaming and micro turbulence is taken place for the implosion of these bubbles which create strong local forces. Mechanical stresses help in releasing proteins from their original matrix. Cell walls, membranes and other structures are broken down by mechanical stress [68]. Ultrasound helps to extract proteins by breaking down cell walls and membranes, which includes those from plant tissues, microbes or animal by products. This turmoil makes it simpler for the proteins to get into the nearby extraction media or solvent. The proteins that are released can be gathered through filtration or through centrifugation. Ultrasound-assisted extraction is faster, provides higher yields of extraction and potentially protects better in comparison with usual techniques for extraction. In regard of the functional characteristics of protein and lesser usage of harsh chemicals, Ultrasound-assisted extraction can be considered as environmentally friendly.

**Fig. 1:**
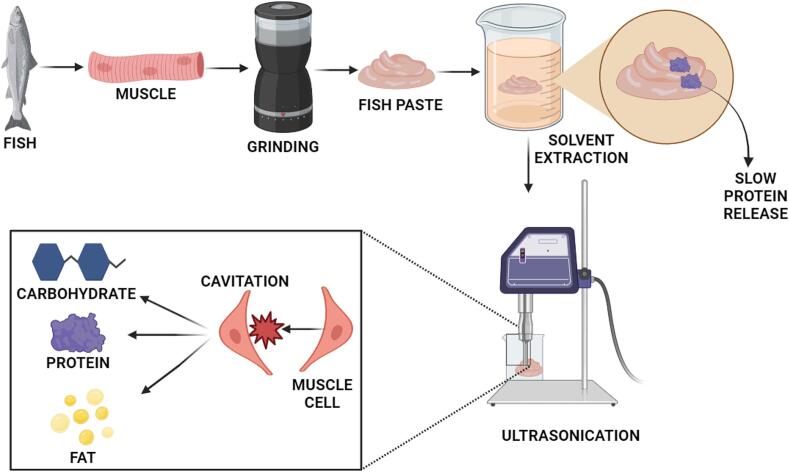
Mechanism of ultrasound for modification of muscle and facilitate protein extraction.

Many factors, influence how effectively proteins are separated from a mixture such as the frequency and amplitude of ultrasound, time given for exposure, nature of sample being treated with ultrasound waves and the choice in solvent used for extraction. To ensure best possible recovery of protein while maintaining its integrity and function, these criteria need to be optimized. Additionally, the time required for extracting proteins is often reduced by using ultrasonic treatments. The ultrasound waves make cavitations and micro streaming which speed up the diffusion of solvents into the biological material, leading to quick extraction kinetics. The capacity to reduce processing time is particularly important in industrial situations where faster extraction methods can enhance production and save money [67]. Lower temperatures along with minimal time extracting conditions are often need for Ultrasound-assisted extraction than the other methods. The procedure assists to maintain the bioactivity, functionality, and denaturation of isolated proteins. This results as the reduction of solvents and energy usage, thus making the process friendly towards environment. Though extraction with ultrasound has multiple benefits, its effectiveness may vary based on the protein sources as well as ultrasound parameters including frequency, chosen extraction solvent and intensity. It is crucial to adjust these parameters correctly for getting the required extraction efficiency and maintaining the protein quality [69]. Henceforth, Fig. 1 depicts the ultrasound technique mechanism for facilitating protein extraction.

In a nutshell, the proteins in the blue foods are structural moieties of the cells combined with other macromolecules such as lipids and glycogen in both plant and animal based blue foods. This structural integration makes it difficult to extract these proteins. The ultrasound pretreatment involves the exposure of the cellular matrix to the high intensity sound waves which in turn disrupt the cellular membrane and releasing the proteins, lipids and other compositional contents. This pre-treatment offers the advantages of shorter extraction time, higher yield and reduced dependency on the chemical reagent for protein extraction making it environmentally friendly and a sustainable processing alternative. However, for effective utilization of the technique, the processing parameters need to be meticulously optimized through experimentation to achieve maximum efficacy and protein functionality.

## 4 Effect of ultrasound on protein modification

Through many alternatives, sound waves known as ultrasound which is a form of mechanical energy can change the molecules of protein. Protein solutions might experience conformational alteration due to the application of ultrasound, in content with molecules by changing their structure and function. Due to energy produced by ultrasonic vibrations, the weak non-covalent bond may get interrupted. The non-covalent bond binds protein together in their original composition including their hydro-phobic nature and the interactions occurring within hydrogen molecules. Thus, this disturbance could lead to denaturation, which is the unfolding of a protein and the hidden hydrophobic areas gets exposed out. Recent research reveals that method can change the proteins' secondary, tertiary and quaternary structures, affecting their enzymatic activities and structural stability [70]. Furthermore, ultrasound has the ability to create cavitations in a liquid medium. Shear forces made by the collapsing bubbles may lead to breaking up of protein molecules which is further influenced by high temperatures and pressures. A method known as sonication is used to break down bigger protein complexes into the smaller ones or even into the individual amino acids. Additionally, ultrasound can enhance mass transfer through acoustic streaming. The streaming modifies the proteins microenvironment and controls the spreading of solute throughout, affecting proteins’ behaviour [71].

The alterations occurred within protein molecules by ultrasound have been used in various strata including processing of food products, medicines, cosmetology and biotechnology. For instance, methods supported by ultrasound can be useful in the food industry. The proteins can be extracted through different sources, enhancing the health benefits such as improving digestion and producing functional products. Altering carriers that are protein-based to boost characteristics of drug encapsulation and release has led to a study on ultrasound for use in drug delivery systems. The possibilities of ultrasound are likely promising; still its impact on protein molecules is intricate. Numerous factors are involved to determine the stability of protein molecules which includes the protein’s exposure time along with its length, intensity and frequency [72]. Thus, working on protein molecules require specific measures to be adjusted carefully in order to avoid denaturation, fragmentation and other alterations in proteins caused by ultrasound method. Understanding the measures of ultrasonication and its impact on proteins is crucial in order to fully exploit its potential while ensuring protein’s preservation, functionality and cellular activity across different sectors [73].

Proteins can get affected by the method of ultrasound in different ways, causing alterations in their structural stability and function. Formation of bubbles and their unwanted explosion under extreme scenarios can be due to acoustic cavitations also known as ultrasonic oscillations. The condition where bubbles burst, the mechanical forces get activated with subject to solution under shear stress along with the occurrence of shock waves, high temperatures, and pressures. The protein structure might undergo alterations due to these shock occurrences, which could result in denaturation. Another reason for such fragment disruptions could be due to the instability of protein’s original structure which could be further due to the disturbance in the adherence of weak non-covalent bonds [74]. In a direct approach, ultrasound can influence the change in the conformation of protein molecules in content with the shear stress impacting the molecules mechanically. The shear stress leads to the unfolding of proteins under the mechanical influence, which reveals out the hidden hydrophobic areas and changes the entire structure of proteins. These changes influence the protein stability and suppress the further enzymatic activity. In addition, due to the shear stress, bigger protein structures might get broken down into smaller or singular amino acids [75].

Furthermore, the heat effects caused by ultrasound play a crucial role in proteins structural and mechanical changes. Even the heat when not generated through ultrasound, the sudden rise in temperature through cavitations might influence the stability of the protein. Changes in temperature could trigger shifts within the structure and function of proteins, leading to denaturation and also conformational alterations. A recent study emphasized the importance of understanding the effects of heat on protein stability and the effect on protein by controlling the temperature during the ultrasound method. The study reveals that the heat impacts, acoustic cavitations and the shear stresses are the primary mechanisms by which ultrasound alter the proteins [76]. Each of the methods can possibly induce alterations in protein structure. As a result, this may have an impact on their functional and mechanical effectiveness. Understanding these pathways is crucial for utilizing ultrasound optimally in multiple areas and achieving the intended changes in the stability of protein molecules [72]. Henceforth, numerous blue food proteins which are subjected to US along with its effect are stated in Table 2.

**Table 2:** Effect of US on the protein from different sources.

Source of Protein	Type of protein	Essential amino	Protein digestibility	PDCAAS value	Reference
Yellow stripes cad	Fish protein hydrolysate (FPH)	Histidine, Isoleucine, Leucine	>90 %	<1	[77]
Atlantic bonito	Complete protein	Glutamic acid, Aspartic acid	90 %	1	[78]
Catfish	Lean protein	Lysine, Isoleucine, Leucine, Valine, Glutamic acid, Aspartic acid, Alanine, Arginine.	80–90 %	92 %	[79]
Whiteleg shrimp (*Litopenaeusvannamei)*	Alpha-amino acids	Arginine, Histamine, Isoleucine, Leucine, Methionine, Phenylalanine, Tryptophan, Lysine, Valine.	85 %	1	[80]
Fish gelatin	Collagen	Glycine, proline, and hydroxyproline	98.8 %	0	[81]
Squid (*Dosidicusgigas)*	Complete protein	Threonine, Arginine, Methionine, Valine, Phenylalanine, Leucine, Isoleucine, Lysine, Histidine	90–95 %	1	[82]
Cod	Lean protein	Lysine, Leucine, Methionine	90–95 %	0.96	[82]
Algae *(Ascophyllumnodosum)*	lectins and phycobiliproteins	Leucine, arginine, and lysine	82 %	<1	[83]
Microalgae (*Scenedesmus* sp.)	lectins and phycobiliproteins	Leucine, Arginine, Lysine, Isoleucine, Phenylalanine, Threonine, Valine.	51–90 %	0.3–0.8	[84]
Diatoms (*Nitzschia* sp.)	Silaffins, Cingulins,	Glutamic acid, Aspartic acid	82 %	0.98	[85]
*Chlorella vulgaricus*	phycocyanin, peptides	leucine, arginine, and lysine	51 ± 9 %	64 %	[86]
Lobster (*Jasusedwardsii)*	Lean protein	Threonine, Leucine, Valine, Lysine, Arginine.	65–86 %	0.95	[87]
Scallops (*Patinopectenyessoensis*)	Lean protein	Glycine, Arginine	86.3 %	67 %	[88], [89]

### 4.1 Factors affecting protein modification

The modification of protein through ultrasound application is attaining wide attention in content with it providing an altered-induced result in proteins’ physical and chemical characteristics. Many factors influence the rate of modifying proteins using ultrasound, but the three most important ones are the exposure time, its power intensity along with the frequency. Changes that are more significant can be attained with greater power intensities, but an excessive use may result in protein denaturation [90]. With the attainment of higher power intensities, correct frequency must be applied to avoid undesirable damage on protein as they are inter-related with severe modifications due to bigger cavitations. Another important element is the time of exposure, as the enhanced modifications could be attained through long exposure but only to a point, beyond the extended point exposure might lead to protein degradation. The alteration caused by ultrasound depends largely on the protein type and its constituents. Thus, the molecular size, structural composition and enzyme involved in the proteins influence the type of modification a protein can attain [91].

Proteins that are smaller could go through bigger changes as they have more surface area to be exposed. The changes found in the protein structures and their bonds like disulfides or certain amino acid residues may become more susceptible to alteration. Moreover, the composition of the surrounding medium and any additives or solvents present could also impact how efficiently ultrasound causes alteration. The process is also highly affected by the factors such as pH, temperature and type of buffer used for reaction [92]. Additionally, modification in protein structure can be greatly influenced by the kind of reactor and structure of ultrasonic is used. The size, geometry and cavitation-promoting surfaces in a reactor are very important factors which determine the efficiency of protein modifications. Moreover, the way ultrasound procedure is arranged and controlled can also impact the results of alteration. This includes factors such as whether continuous or pulsed ultrasound is applied, which have not been studied much. A study from 2020 emphasized on the effect of power intensity and the frequency in sono-chemical effects during the protein modification [93]. Furthermore, Rahman & Lamsal, (2021) focused on the influence of protein composition and structure in causing alterations undergoing the ultrasound process. Understanding these elements is crucial for improving ultrasonic methods related to protein changes in order to obtain specific and controlled modifications of proteins [90].

### 4.2 Pre-treatments for protein modification

The methods to alter the protein structures, are determined through the type of pre-treatment been done. For the implementation of modification, the major concern is to turn protein structures in more appropriate and in an easier way to modify later. In order to achieve stable and accurate protein modification, different types of pre-treatment methods have been studied − each carries its own effect on the structure of protein. One of the major pre-treatments is based on enzymes which includes Proteolytic enzymes. These enzymes break certain peptide bonds or influence the change in structure of proteins for the alterations through turning the target regions more attainable. As per the latest study, methods of enzymatic pre-treatment of proteases in content with further modification have shown efficient result in altering protein conformation. The study of 2022 revealed the same features that to achieve required modifications, boosting up the usage of enzymatic pre-treatment is crucial [94]. Chemical based pre-treatments influences the change in the structure of proteins directly or by reacting on the disulfide coupling by using solvents and other reducing agents. This leads to rise in the number of reactive sites being exposed for the later alterations. One of the researches from 2015 exposes that chemical denaturation plays an important role in pre-treatment, turning the ensuing protein changes more effective [95].

According to Lee et al., (2022)’s research, chemicals such as urea or guanidine hydrochloride (chaotropic) influences the unfolding of protein structures [74]. This assists in exposing buried residues which might be difficult to access otherwise by causing them to become exposed more readily. To attain increased susceptibility for further changes can be achieved through physical treatments. This treatment leads through the alteration made to protein structure by using physical stimuli including the heat receptors, pressure and techniques like ultrasound. For instance, using heat and pressure might lead to proteins unfolding and resulting in conformational alterations, respectively. According to Wang et al., (2021) ultrasound as a physical pre-treatment can modify the reactive sites to exceed its accessibility by changing the protein structures [96]. Compiling the pre-treatments together mainly enzyme, chemical as well as physical methods have shown high potential and enhanced the efficiency along with specificity for better protein modifications. These combined techniques are designed to change the protein structures in a collaborative way, upgrading the exposure of desired sites for additional modifications. Another study indicated that applying different pre-treatments improved the protein's structure to enable further changes. The adaptation methods for proteins require customization and optimization, considering the type of pre-treatment techniques and its effect on protein structure. These techniques of protein based pre-treatment provide pathways for regulation and precise changes, resulting in the desirable characteristics [90].

In conclusion, Ultrasound, a form of mechanical energy, can alter the protein molecules by disrupting sulphur and hydrogen bonds and causing changes to secondary, tertiary and quaternary structures. This leads to protein denaturation where some of the hydrophobic region get exposed impacting the proteins functions. The process is driven by ultrasonic cavitation where collapse of bubbles generates very high-shear forces, localised temperature, and pressure leading to protein fragmentation. This mechanism led to conversion of large protein complexes into smaller peptides with variable functional properties. Optimising ultrasound parameters can allow controlled and specific protein modification making ultrasound a valuable treatment for protein modification. The effectiveness of ultrasound treatment can be enhanced by enzymatic or chemical modification by making target site more accessible. Enzymes like protease break the peptide bonds while chemicals like organic acids helps in the unfolding of protein structure. Physical pre-treatment such as heat and pressure can also lead to confirmational changes.

## 5 Bioactive peptides from blue food proteins

Many health advantages are achieved from bioactive peptides which are extracted through different modes from blue food proteins. These proteins are originated from specific functional groups they consist of. The bioactive compounds usually consist of small amino acid chains. The main characteristic of these peptides is that they hold excellent antioxidant activity. This activity leads to scavenge off the free radicals and inhibit the reactive oxygen species (ROS) activity, helping in the reduction of oxidative stress and controlling the damage within the cells [97]. These bioactive peptides contain peptide bonds, sulphur containing compounds and aromatic rings (phenylalanine and tyrosine) as their functional groups. These functional groups help to neutralize the free radicals effectively, which assists in protecting cells from oxidative damage. The bioactive peptides from blue food proteins also have promising anti-hypertensive effects. They function via the renin-angiotensin activity, particularly angiotensin-converting enzyme (ACE). These peptides could potentially contribute to blood pressure regulation by preventing the conversion of angiotensin-II from angiotensin-I through their inhibition of ACE [98].

The sequences of these peptides that brings out this inhibition, includes proline and hydrophobic amino acid residues. They resemble similar structure as of ACE inhibitors and are in competitive interaction with the ACE active site. This lead to hinder their enzymatic activity and further helps to control the blood pressure. In addition, the binding of blue food-bioactive peptides with receptors are proven to aid in managing hunger and promoting a feeling of fullness by managing the hormone such as leptin and ghrelin [99]. These receptors get vitalize by these peptides bonds because they impersonate the form of these hormones which could potentially result in an effect related to satiety. This effect on satiety might have some implications for controlling the intake of food. This leads to assist in the methods for dietary control and managing weight. Additionally, the bioactive peptides from blue food proteins have shown effects against diabetes by affecting glucose metabolism. For instance, peptides from marine sources could hinder the actions of enzymes that break down α-amylase and α-glucosidase (carbohydrates) [100]. These sea-derived peptides possess numerous amino acids and their sequences which blocks the active site of these enzymes. This sequence slows down the carbohydrate breakdown and glucose absorption leading to improve the glycaemic activity control.

**Fig. 2:**
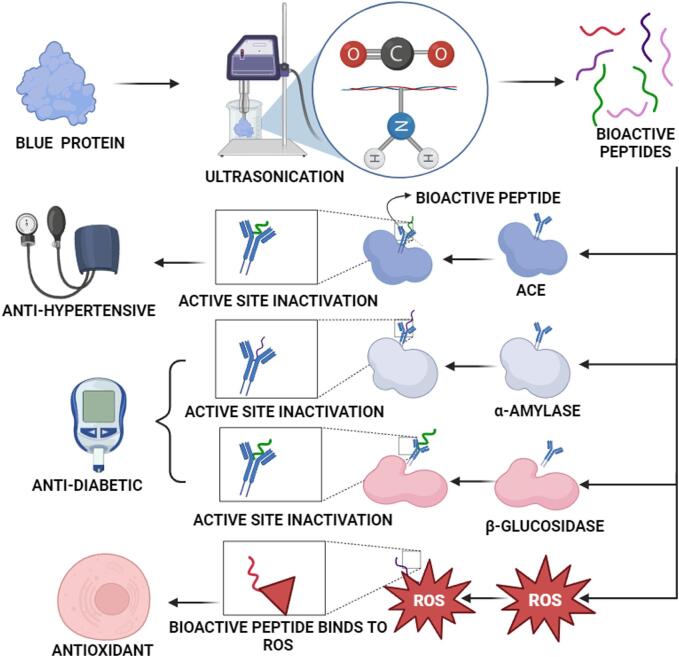
Mechanism of bioactive peptides for different bioactivity.

The methods of these bioactive peptides are quite complex. This includes the functional groups, enzymes, receptors and hormones present within these peptides and interactions between them. For example, the aromatic rings and certain amino acids helps these peptides scavenge off free radicals and turn the targeted enzymes activated for the interactions, respectively. This inter-connection plays a vital role in the bioactivities which permit their intricate modes of action to give various health benefits. In result, the explorations of these bioactive peptides found in blue food proteins have effective advantages on human health and wellness [101]. The study of 2021 reveals that to comprehend the complex linkages between the peptides and their biological traits are crucial. The importance of their precise sequences, as well as potential uses in functional foods or medicine is quite benefit for the industries. Thus, to conduct more researches to fully comprehend all these bioactivities and their interactions are important. For the enhanced therapeutic advantages, these bioactive peptides are to be safeguarded to be incorporated in different health-promoting products. They offer many possible health benefits, showing potential in the nutrition fields such as food-based products and medicines. The study of 2020 aimed to explore the continued research efforts for discovering the natural algal products and their therapeutic advantages which can be provided to human health [102]. Thus, Fig. 2 depicts different bioactive peptides activity and functionality.

## 6 Potential for commercial application

In the field of food science and technology, wide range of unexplored nutrient traits are still hidden and often overlooked. One of the highly promising groups includes the blue food proteins. This term encompasses many natural sources like blue-green algae and well-known items such as blueberries, butterfly pea blooms along with various fish types consisting blue pigments. Recently, the food industry is recognising these blue foods and their potential because they consist of rich nutrients, lively natural pigments and the nutraceutical benefits [103]. Compounds obtained from blue food could be good for health due the presence of various bioactive substances. For instance, Anthocyanin rich blueberries are famous as a strong antioxidant. These are related to regulate the cardiovascular as well as cognitive health of an individual. Foods like spirulina and chlorella, which are algae present in the blue-green color, contain high protein content, minerals, vitamin sources and crucial fatty-acids. They might be useful to tackle malnutrition while offering sustainable food variety. In different sectors, the possibility of making money from blue foods is already showing itself [104].

Blue spirulina, also known as spirulina algae, is a natural colorant that can be included in the food items and drinks without requiring artificial additives. Natural addition has led beverage firms to use the blue pigment in their drinks manufacturing. This latest trend to create attractive products that focuses on health benefit aligns with the increasing desire for natural elements among customers is gaining interest. Furthermore, the nutraceuticals industry has turned towards blue foods due to the presence of numerous bioactive compounds. The separation and compression activity of these bioactive compounds to enhance the functional foods and produce dietary supplements is called nutraceuticals developed with blue food sources [105]. One of the examples of this activity is blue-green algae, because it contains many nutrients which act as an encouraging avenue for nutraceuticals development. Experts are examining the potential benefits through extraction from these algae on the inflammation reduction, anti-oxidation and boosting the immunity system. The field of nutraceuticals are recognizing the possible advantages of these substances in addressing present health problems. This understanding has encouraged studies on potential combinations to be used in the functional foods as well as the food based supplements [91].

For using blue food sources in the commercial nutraceutical products, the major obstacle found are from the standardization and quality control of blue foods. The effectiveness of bioactive substances is linked to the concentration consistency of chemicals within each batch. To control the compound bioactivity in nutraceuticals applications, one of the crucial steps is to conduct thorough research for deciding on the best techniques for extraction and formulations for the better development. With the increasing functional needs for natural and sustainable foods, there is great potential in using blue foods for commercial purposes. Still, more studies and investigation to formulate this research are needed to bring this development to reality [106]. Food tech researchers, scientists and nutraceuticals industry can work together to fully exploit the potential use of blue foods for creating innovative products that promote health. The link between commercial activities and nutraceuticals promotes an optimistic synergy to exploit the bioactive components found in blue foods as natural products that improves health. The combination of scientific advancement and the abundance of nature forms a novel and intriguing step in the ongoing research for blue foods that promote sustainable and healthy eating habits [107].

## 7 Future prospects

Ultrasound, known for its non-intrusive and adaptable nature, demonstrates significant potential across multiple commercial sectors, notably in medicine, industry, and research. While commonly utilized for imaging, diagnostics, and therapeutic interventions, its versatility extends far beyond these realms. Conventional (solvent and alkali-based) protein extraction methods, have limitations and are not suitable for sensitive proteins or large-scale applications. These may create lack of specificity and lower the extraction yields due to protein degradation. UAE is one of the best physical extraction methods for protein as it can extract bioactive components in very less time, at low temperature, with lesser energy and solvent requirement [108]. Also, being a non-thermal technique, it is better equipped to retain the functionality of the bioactive compounds [109]. US treatment, as a safe, non-toxic, effective, and non-polluting treatment method, has been widely used to improve protein’s structure [110]. Moreover, US is energy efficient, easy to install, and has low maintenance costs. In comparison with other eco-innovative technologies, US requires low investment and shorter extraction times, whereas ohmic heating requires a medium investment with medium extraction times, and continuous pulsed electric field requires high investment and medium extraction times [111], making it more efficient for application in coming future [111].

According to Gallo et al. (2018), this technology can replace traditional sanitization methods and upgrade hygiene status and overall quality of the food product, provides a higher degree of safety. An increasing number of industrial processes utilize US to aid in the process of mixing materials, form foams and agglomerates, precipitate dust, improve filtration efficiency, dry products, and extract solid materials and bioactive compounds from vegetables and food [112]. US has great potential to be used for commercial application due to these above-mentioned reasons that how it replaces the traditional technologies in a diversity of sectors and serves better in place with more efficiency.

Khan et al., (2021) concluded in one of the studies that the US treatment changed the structural and functional properties of sea buckthorn seed protein concentrate. The US treatment changes the secondary structure (% of α helix and β-sheets), particle size and surface hydrophobicity which directly enhanced the solubility and emulsifying activity index (EAI). Now, this occurs due to disruption of non-covalent bonds, resulting in unfolding of protein molecules and exposure of hydrophobic regions, which facilitates better interaction with solvent and emulsion stabilization [113].

US has subsequent effects on the microstructure of protein; In addition, protein isolate or concentrate can also be treated with ultrasonication to influence protein microstructure [90]. Utilizing US proves effective in boosting extraction processes, particularly with the utilization of low boiling point solvents, while maintaining the extraction mixture's temperature below its boiling point. The ultrasonic cavitations lead to the production of shear waves, microjets, turbulence and shock waves which enhances the extraction by modifying the plant matrix [114]. These intense yet localized forces help disrupt cell walls and membranes, facilitating the release of proteins without the need for harsh solvents or excessive heat. Modification of the protein structure and hydrophilic residue formation, leads to a decrease in protein molecular weight and an increase in the interaction between protein and water molecules [115], [116]. This gentle extraction process preserves the structural integrity and biological activity of the extracted proteins, making them more suitable for various applications in the food, pharmaceutical, and biotechnology industries [117]. It reduces the hypothermal effect, which subsequently diminishes the degradation or denaturation of protein and as a result, extracts high-quality protein (Kumar et al., 2021).

The protein requirements for athletic populations have been the subject of much scientific debate for both strength/power and endurance. High protein diets have also become quite popular in the general population as part of many weight reduction programs. Another case is the rising rates of obesity and diabetes, rising eye-health concerns, increase in cardiovascular disease incidence, transforming consumption patterns in developing markets, rising preference for preventive medicine, rising popularity of nutraceuticals with organic and natural ingredients, and strong demand for multivitamins and other ingredients such as omega 3 s and astaxanthin are factors propelling the nutraceuticals and functional food market [118]. Nutraceuticals containing ingredients such as conjugated linoleic acid (CLA) soy, whey, and dietary fibres with claims of weight management, heart care, immunity, and digestive health are gaining popularity [119]. Products containing polyphenols have especially very high sales, particularly in cardiovascular conditions [120], Carotenoids and curcuminoid-containing nutraceuticals, astaxanthin [AX], turmeric [121], [122]are also well-represented in the top selling product, and herbs from TCM and Ayurveda are entering the mainstream global market. The utilization of bioactive peptides in the development of nutraceuticals involves incorporating these peptides into functional foods, supplements, or pharmaceutical products, thereby enhancing their health-promoting properties [123]. In particular, marine peptides have attracted a great deal of attention due to their potential effects in promoting health and reducing disease risk. These peptides have been obtained from algae, fish, mollusc, crustacean, and marine by-products including substandard muscles, viscera, skins, trimmings and shellfish. Marine bioactive peptides based on their structural properties, amino acid composition and sequences have been shown to display a wide range of biological functions including antioxidant, anti-hypertensive, antimicrobial, opioid agonistic, immunomodulatory, prebiotic, mineral binding, anti-thrombotic and hypocholesterolaemia effects [124].

According to the European Food Information council, functional foods must contain biologically active components that have the potential to optimize physical and mental well-being and which may also reduce the risk of disease. Such foods include fortified food, enhanced food, enriched food, dietary supplements, and health food [119]. The development and application of bioactive peptides in nutraceuticals will probably play a crucial role in satisfying consumer demand for natural, functional, and health-enhancing products as it continues to rise. The result will foster a healthier and more proactive strategy for maintaining personal health and wellness [107]. Furthermore, the use of US in protein modification is becoming more popular. This is the process of changing the characteristics of proteins for particular industrial or medicinal uses [73]. The utilization of US within the food sector is anticipated to proliferate, underpinned by escalating consumer demand for functional foods and nutraceuticals, driven by heightened health consciousness. UAE is poised to assume a pivotal role in the fabrication of functional food constituents, including bioactive peptides and marine peptides, renowned for their health-promoting attributes. Furthermore, ongoing innovations in US technology are projected to refine its efficacy, precision, and versatility. Advances in equipment design and engineering are expected to yield specialized US systems finely tuned to address specific applications within diverse industrial domains.

## 8 Conclusion

As our world population continues to rise, so does the demand for nutritious food sources, particularly those rich in protein. This trend underscores the critical importance of addressing food security challenges and finding sustainable solutions to meet the nutritional needs of billions of people worldwide.

While plant-based proteins offer several benefits, they also come with limitations that render them less optimal compared to animal-based sources in certain contexts. Several reasons contribute to this assertion, namely, incomplete protein profile, lower protein digestibility and limited nutrient density. The nine EAAs are especially important, as human cells cannot synthesize these at sufficient rates to meet metabolic demand, and therefore humans must obtain them through their diet. PBPs (Plant-based proteins) have relatively low amounts of EAAs and leucine contents compared to ABPs (Animal-based proteins), even some PBPs are low in lysine, cysteine, and/or methionine. So, one may need to consume in large quantities to attain sufficient amount of protein to meet metabolic demands for muscle synthesis. It also contains some Anti-nutritional factors like phytates and oxalates, which can interfere with the absorption of several minerals like iron and calcium. Due to the potential nutrition, we may require careful planning to ensure you get all the essential nutrients, including vitamin B12, vitamin D, iron, and omega-3 fatty acids. Fortified foods or supplements may be necessary for some nutrients. With the increased usage of PBPs in many food categories, consumers with allergies, and even individuals that may not have experienced a previous allergic reaction, need to be cautious. For example, individuals with soy or peanut allergies must be especially cautious of foods containing pulse proteins. Some individuals with wheat intolerance or allergy may not be aware they will also negatively respond to barley and rye proteins.

Now in contrast to this, the significance of blue foods as a protein source cannot be overstated, particularly in the context of global food security and sustainability. Blue foods, encompassing a diverse array of aquatic resources such as fish, shellfish, algae and some crustaceans offer several compelling advantages, for instance, cultural culinary diversity, resilience to climate change, and blue foods are also rich in high-quality protein, EAAs, vitamins (including B12 and D), minerals (such as iodine, iron, and zinc), and omega-3 fatty acids. These nutrients are vital for supporting overall health, cognitive function, and immune system strength.

Ultrasound technology holds immense promise for enhancing protein preservation, improving quality, and increasing bioavailability, while also enhancing bioactivity in various food products, it effectively inhibits microbial growth and enzyme activity in protein-rich foods and increases the shelf-life. It can disrupt the structural barriers within protein matrices, increasing the accessibility of proteins to digestive enzymes and facilitating their digestion and absorption in the gastrointestinal tract. This enhances the bioavailability of EAAs and other nutrients, promoting better nutritional outcomes and metabolic health. Embracing blue foods as a protein source offers a promising avenue for addressing global nutrition challenges. By harnessing the nutritional richness of marine resources like fish, seaweed, and algae, we can enhance protein intake while mitigating environmental pressures associated with land-based protein production. Through targeted investments in research, infrastructure, and consumer awareness, integrating blue foods into dietary patterns can foster healthier communities and promote sustainable food systems for generations to come. This convergence of nutritional potential and technological advancement highlights the promising future of blue foods as a valuable protein source, addressing the nutritional needs of an ever-growing global population.

## CRediT authorship contribution statement

**Ghumika Pandita:** Writing – review & editing, Writing – original draft, Formal analysis, Conceptualization. **Savvy Sharma:** Writing – review & editing, Writing – original draft, Visualization, Supervision, Formal analysis, Data curation, Conceptualization. **Irin Elsa Oommen:** Writing – review & editing, Writing – original draft, Methodology, Investigation, Data curation, Conceptualization. **Nishchhal Madaan:** Writing – review & editing, Visualization, Validation, Software, Formal analysis, Data curation, Conceptualization. **Yuvraj Bhosale:** Writing – review & editing, Visualization, Validation, Supervision, Project administration, Methodology, Conceptualization. **Vivien Nagy:** Writing – review & editing, Visualization, Software, Resources, Methodology, Formal analysis. **Ayaz Mukarram Shaikh:** Writing – review & editing, Visualization, Validation, Software, Formal analysis, Conceptualization. **Béla Kovács:** Writing – review & editing, Validation, Supervision, Resources, Project administration, Investigation, Funding acquisition, Conceptualization.

## Declaration of competing interest

The authors declare that they have no known competing financial interests or personal relationships that could have appeared to influence the work reported in this paper.
